# Kikuchi-Fujimoto Disease in the Unites States: Three Case Reports and Review of the Literature

**DOI:** 10.4084/MJHID.2014.001

**Published:** 2014-01-01

**Authors:** Darcie Deaver, Mojdeh Naghashpour, Lubomir Sokol

**Affiliations:** 1 Department of Malignant Hematology, H. Lee Moffitt Cancer Center and Research Institute, Tampa, FL; 2 Department of Hematopathology, H. Lee Moffitt Cancer Center and Research Institute, Tampa, FL

## Abstract

Kikuchi-Fujimoto Disease (KFD), also known as histiocytic lymphadenitis, is a benign, self-limiting disease that manifests primarily as cervical lymphadenopathy but may include low-grade fever, headache, and fatigue. There is a higher incidence of KFD in women aged 20–35 years and in Asian populations. A PubMed search revealed 590 articles that described KFD. Of these, 22 cases have been fully described in the United States. Ten of the 22 (45%) patients were male and 12 (55%) were female, with 20% Caucasian, 20% Asian American, and the remaining 60% of other ethnic backgrounds. In this study, we describe an additional 3 cases of KFD and discuss the diagnosis, pathology, and management of KFD.

## Introduction

Kikuchi-Fujimoto Disease (KFD), also known as histiocytic necrotizing lymphadenitis, was first described independently in 1972 by Kikuchi and Fujimoto.^1^,^2^ The initial presentation of KFD includes cervical lymphadenopathy, low-grade fever, headache, and fatigue.^3^,^4^ Other less common symptoms include nausea, vomiting, night sweats, and weight loss. Cutaneous manifestations may also be a presenting sign; however, this is a less common finding.^4^ Laboratory studies may reveal leukopenia, anemia, elevated erythrocyte sedimentation rate, and elevated C-reactive protein.^3^ Atypical lymphocytes may also be present in the peripheral blood.^4^

A recent PubMed search of articles published between 1972 and January 2013, revealed 734 articles that have described KFD. Keywords used included “Kikuchi-Fujimoto disease” and “histiocytic necrotizing lymphadenitis.” Dorfman and Berry evaluated 108 cases with a confirmed diagnosis of KFD.^5^ Of these, 88 were diagnosed in the United States. This article provided gender, age, and ethnic information for the total population studied but did not stratify the information for patients in the United States alone. In addition, for our search, those articles that did not provide the ethnicity, age, and gender of the patients studied were not included in our review. We focused our review on the adult population although it is known that KFD may also occur in children.^6^ A total of 22 cases with KFD have been fully described in the literature.^7^–^24^ In this report, we describe 3 patients diagnosed with KFD and discuss the diagnosis, pathology, and management of this rare disease.

## Case Presentation

### Patient 1

**Figure 1:**
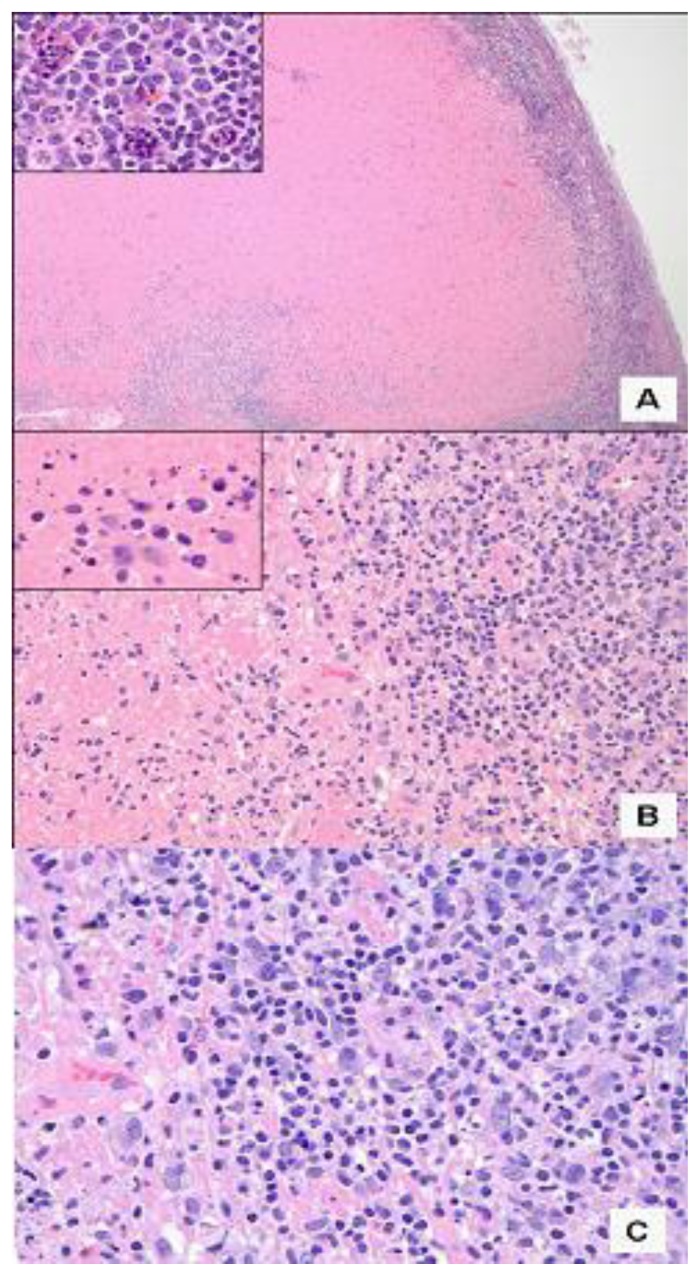
Kikuchi lymphadenopathy of a cervical lymph node. A) Prominent tissue necrosis with extensive apoptosis, mostly of histiocytes (inset) (hematoxylin and eosin; ×40). B) Fibrinoid necrosis with abundant nuclear fragmentation (inset) but no leukocytic inflammatory infiltrate (hematoxyline and eosin; ×200). C) Sheets of proliferating histiocytes (hematoxylin and eosin; ×400).

Patient 1, a 26-year-old white female, presented in February 2008 with approximately four enlarged lymph nodes in the left posterior cervical area. She also complained of joint pain and fever that lasted approximately 2 weeks. Laboratory data were reportedly normal. She was initially diagnosed with infectious mononucleosis. She again presented 2 weeks later complaining of cervical lymphadenopathy and concurrent joint pain. Laboratory data at that time revealed leukopenia and thrombocytopenia. She was subsequently referred to a hematologist. She was hospitalized and underwent a comprehensive work up, with tests revealing a white blood cell count of 2.0 (10^3^/mm^3^), with a total lymphocyte count of 900 cells/μL and total granulocyte count of 1000/μL, hemoglobin level of 12.9 g/dL, mean cell volume of 91.5 fL, and platelet count of 100,000/μL. HIV-1 and −2 screening tests were non-reactive, and anti-DNA and anti-nuclear antibodies were negative. Epstein-Barr Virus (EBV) and cytomegalovirus (CMV) tests were negative. Bone marrow biopsy was without evidence of malignancy and showed normal cytogenetics (46, XX). Chest X-ray suggested chronic bronchitis with minimal peribronchial thickening. CT scan of the abdomen and pelvis without contrast revealed mild hepatosplenomegaly, with the spleen measuring at least 17 cm in length. Results also suggested some dilation of the portal venous system, as well as bibasilar infiltrates with a small amount of pleural reaction. A repeat chest X-ray confirmed the interval development of bibasilar infiltrates that were worse on the left side, suggesting pneumonia. At that time, she was treated with intravenous antibiotics. Follow-up chest X-ray showed no active infiltrates. No adenopathy was noted in the chest on any study that was performed. PET/CT scan showed hypermetabolic adenopathy bilaterally in the neck, left supraclavicular, and left subclavian areas. The largest lymph node measured 2.4 cm in the anterior triangle of the left neck. The SUV maximums ranged from 3.7 to 9.8. There was no hyper-metabolism in the axillary, mesenteric, retroperitoneal, or pelvic areas. Patient 1 underwent an excisional biopsy of a left cervical lymph node. The initial differential diagnosis considered aggressive lymphoma, and she was recommended to start chemotherapy. However, she was referred for a second biopsy, and the diagnosis of KFD was suggested. Flow cytometry performed on the lymph node specimen did not identify a clonal B-cell population. The lymph node biopsy slides were reviewed at our institution. A section of the node showed extensive geographic area of cell necrosis with a rim of histiocytes and immunoblasts. Frequent single cell apoptosis was noted. Rare neutrophils were identified. Frequent plasma cells and plasmacytoid monocytes were also noted (Figure 1). These features were consistent with the diagnosis of KFD. Spontaneous resolution of the lymphadenopathy occurred within 4 months of diagnosis.

### Patient 2

Patient 2, a 21-year-old female, presented with a 1-year history of generalized achiness, chronic sinus infections, chronic mouth ulcerations, and recurrent strep infections that required treatment with multiple antibiotics. She later developed mesogastric pain. Physical examination demonstrated abdominal tenderness with palpation. CT scan revealed mesenteric adenopathy. She was then referred to a surgeon for laparoscopic biopsy of an accessible lymph node. Upon evaluation by the surgeon, she had new onset epigastric pain, fatigue, and night sweats that were getting progressively worse. She also had a 3-week history of diarrhea, stomach upset, cough, and pain after urination. Labs at that time revealed white blood cell count of 16,000, hemoglobin level of 13.1 g/dL, platelet count of 346,000/μL, polymorphonuclears at 83%, and lymphocytes at 8.7%. Physical examination performed by the surgeon revealed 1 submandibular lymph node on the left side and 1 small (1 cm) cervical lymph node in the right cervical chain. The patient underwent a laparoscopic biopsy, and a 2 × 1.4 × 1.2 cm lymph node was removed. Intraoperatively, the lymph nodes showed diminished cortical follicles with patent sinuses. Initial pathological review suggested aggressive lymphoma. Upon review for diagnostic confirmation, the histology revealed patchy areas of necrosis consisting of brightly eosinophilic fibrinoid deposits, including nuclear fragments and apoptotic bodies, surrounded by large collections of pale-staining histiocytes. At the periphery of the necrotic areas, there were nests of plasmacytoid monocytes and immunoblasts. Neutrophils and eosinophils were absent. The findings supported the diagnosis of KFD. The patient continued to have residual mesenteric adenopathy for approximately 2 years following diagnosis but this has since resolved completely.

### Patient 3

Patient 3, a 33-year-old female, initially presented with a right neck mass approximately 1 year before evaluation. Six months later, she developed persistent night sweats and diffuse pruritus. A fine-needle aspiration of the right supraclavicular mass was completed, and the final diagnosis found an atypical lymphoid hyperplasia with an indeterminate gene rearrangement. The pathology recommended additional biopsy to rule out lymphoproliferative disorder. An open biopsy of the right supraclavicular lymph node was performed. The final pathology report suggested lymphoproliferative disorder. Initial findings included an atypical lymphoid proliferation with necrosis. The immunohistochemistry revealed CD20 positive, CD10 positive, CD3 positive, CD43 positive, and CD30 positive in small clusters of cells and CD15 positive in scattered neutrophils. Flow cytometry found no clonal B-cell population by immunophenotypic criteria. The CD4/CD8 ratio was unremarkable. A PET scan found a single metabolically active right supraclavicular lymph node. Patient 3 underwent comprehensive staging studies, including viral studies on peripheral blood, which were negative, and bone marrow biopsy, which was negative for involvement of lymphoma. She subsequently underwent surgical excision of the solitary lymph node. The findings confirmed KFD. After excision of the lymph node, repeat CT scan was without lymphadenopathy. The patient has exhibited no recurrent lymphadenopathy and all other symptoms resolved.

## Discussion

### Physical Presentation and Diagnostic Findings of KFD

Patients may present with fevers, chills, weight loss, arthalgia, splenomegaly, and skin rash.^23^ However, the most common presenting symptom is localized lymphadenopathy. Most commonly, lymphadenopathy is located in the jugular carotid chain and the posterior cervical triangle, although there may be generalized, diffuse lymphadenopathy.^25^,^26^ Lymph nodes are usually 3 cm or less in diameter; however, they may reach 5–6 cm in diameter.^27^ Additional findings may include increased lactate dehydrogenase, leukopenia, and elevated serum transaminases.^28^ Approximately 25–58% of patients experience leukopenia and 2–5% of patients experience leukocytosis.^29^ Elevations in erythrocyte sedimentation rate have also been noted.^26^ Excisional biopsy of a representative lymph node is the preferred way to obtain a sample of tissue for microscopic evaluation. Skin manifestations have been identified in approximately 30% of KFD patients. These manifestations are non-specific and include acneiform eruptions, facial erythema, indurated, erythemic papules and plaques, purpura, and nodules.^31^,^32^,^33^ Occasionally, patients will experience lip or eyelid edema and oral ulcers.^31^ Biopsy of any of the cutaneous lesions usually reveals leukoclastic vasculitis, superficial and deep lymphohistiocytic perivascular infiltrates with nuclear debris, papillary edema, and absence of neutrophils.^27^,^31^ Skin lesions are indicative of a more severe clinical presentation, such as presence of liver dysfunction.^32^ Histopathologic findings indicative of KFD include partially preserved nodal architecture with expansion of the paracortex by patchy areas of fibrinoid necrosis with marked apoptosis and nuclear debris, surrounded by aggregates of histiocytes with crescentic nuclei, activated T-lymphocytes (immunoblasts), plasmacytoid monocytes, and characteristic absence of neutrophils and eosinophils.^25^,^29^ Plasmacytoid monocytes with interspersed karyorrhexis and crescent-shaped histiocytes have been considered minimum diagnostic criterion for KFD.^30^ Histiocytic proliferation tends to be more characteristic of KFD than necrosis alone. Differential diagnoses that should be considered include lymphoma, systemic lupus erythmatous (SLE), tuberculosis, and infectious mononucleosis. Because there are no diagnostic laboratory studies available for KFD, it is important to exclude other causes of necrotizing lymphadenopathies, as its course and treatment are entirely different.^29^ To assist in narrowing the differential diagnoses, the absence of auto-antibodies including anti nuclear auto-antibodies may help to exclude autoimmune disorders.^25^ In the early proliferation stage of KFD, the presence of clusters of large atypical cells and immunoblasts may mimic lymphoma.^31^ In lymphoma, however, necrosis may or may not be extensive, and neutrophils and granulomata tend to be absent.^31^ Of note, lymphoma is incorrectly diagnosed in approximately 30% of cases of KFD.^29^ Immunohistochemical staining may reveal a predominance of CD8-positive lymphocytes.^30^ These stains assist in differentiating KFD from lymphoma.^3^ In large B-cell lymphoma, neoplastic cells are CD20 positive, whereas the large cells in KFD are CD8-positive immunoblasts and CD68-positive histiocytes.^3^,^26^, ^30^

### Etiology/Pathogenesis of KFD

The exact cause of KFD is unknown; however, it has been hypothesized that KFD may be the result of a viral infection or a manifestation of an autoimmune disease.^25^ Infectious causes have been suspected due to the self-limiting nature of KFD. The presence of reactive histiocytes and tubuloreticular inclusions identified under electron microscopy, in addition to an elevation of antibodies against an antigen, has supported a viral infection as the source of KFD. The tubuloreticular inclusions were also noted in endothelial cells and lymphocytes in patients diagnosed with SLE.^31^ Due to the presence of reactive histiocytes and atypical lymphocytes, viral infections such as Epstein-Barr virus (EBV) and human T lymphocyte virus-1 (HTLV-1) were identified as possible causes of KFD.^34^ In situ hybridization, polymerase chain reaction (PCR), and immunohistochemistry revealed association between HTLV-1 infection and the development of KFD.^34^ Hudnall et al.^35^ evaluated 30 lymph nodes involved by KFD. The results showed that herpes simplex virus 1, varicella zoster virus, and human herpes virus 8 (HHV-8) DNA were not detectable; and herpes simplex virus 2, cytomegalovirus, HHV-6, and HHV-7 were only detected on occasion. Therefore, it is unlikely that these viruses are involved in the pathogenesis of KFD. EBV was detected in many of the KFD samples; however, it is also important to note that recent EBV exposure may lead to positive serologic studies even if there is no active EBV infection.^35^ EBV titers may be elevated in reactive lymphoid tissues and are not indicative of an active infection of EBV.^28^ Elevated viral titers may persist long after resolution of the infection. Truly relevant serologic results include a 4-fold increase in the IgG titer or the detection of an elevated IgM titer occurring during the disease process.^28^ Cho et al.^36^ evaluated 50 lymph node specimens that were affected with KFD for the presence of HHV-6, −7, and −8. In this study, PCR analyses failed to establish a correlation between the presence of HHV and KFD.^36^ Zhang et al.^37^ demonstrated that parvovirus B19 may be implicated in KFD and identified an association between parvovirus infection and KFD. A strong correlation between SLE and KFD has been established. SLE may be associated with fever, arthralgia, and lymphadenopathy, features that are also characteristic of KFD.^25^ Lymphadenopathy has been reported in 23–34% of patients with SLE, and these patients were more likely to have increased SLE disease activity levels.^25^ Lymphadenopathy associated with SLE is widespread, whereas lymphadenopathy associated with KFD is more localized, usually only affecting the cervical lymph nodes. Histologically, lymph nodes evaluated in SLE and KFD are very similar and demonstrate nonspecific changes with the exception of necrosis.^25^, ^31^ The presence of increased vascularity, scattered immunoblasts, plasma cells, and hematoxylin bodies are also indicative of SLE-affected tissue.^25^, ^31^ The presence of hematoxylin bodies is unique to SLE; however, it is rarely observed in affected specimens. Therefore, histopathological findings are not always definitive in establishing a diagnosis. The presence of large numbers of plasma cells encompassing necrotic foci is also histologically indicative of SLE.^29^ Other pathologic findings in patients with SLE may include malar rash, proteinuria, photosensitivity, auto-antibodies, and the presence of anti-native DNA antibodies.^26^, ^27^ There is yet to be an identifiable cause of KFD and its symptoms.

### Treatment of KFD

KFD is a self-limited, benign condition that spontaneously resolves within 1–4 months; however, 3–4% of patients will experience recurrent episodes of KFD.^26^ Due to the correlation between KFD and SLE, patients presenting with KFD should be evaluated for the presence of SLE.^25^ It is also advised that, following a diagnosis of KFD, the patient should be monitored for the development of lupus.^25^,^33^ Treatment is unnecessary and not recommended unless there is coincidence with SLE. For complicated cases of KFD, glucocorticoids, alone or in combination with hydroxychloroquine, may be the treatment of choice.^22^

## Conclusion

This disease usually affects women in the age range of 20–35 years. The female-to-male ratio is approximately 4:1. Also, there is noted to be a higher incidence in Asiatic populations. Of the 22 adult cases described in the United States, ten (45%) were male, and 12 (55%) were female, with ethnicity showing 29% Asian American, 24% African American, 19% Caucasian, 14% Hispanic, and 14% Western Asian. Age ranged from 6 to 63 years with a mean age of 34 years. Initial presentation was typical for most patients (lymphadenopathy, fever, and fatigue, with most experiencing spontaneous resolution of their symptoms within 6 months after diagnosis).^7^–^24^ The patients described in this case report were female with an age range of 20–35 years of age and they presented with clinical characteristics including lymphadenopathy, pain, and sweats. The patient in case number 2 was interesting in that irritable bowel syndrome (IBS) could have been included in her initial differential diagnosis due to the severe abdominal pain, diarrhea, and upset stomach that she experienced. Imaging studies revealed mesenteric adenopathy that would not be expected with IBS. Furthermore, lymph node biopsy solidified the diagnosis of KFD.

For patient 1, spontaneous resolution of lymphadenopathy occurred within 4 months of diagnosis. Patient 2 continued to have residual mesenteric adenopathy for approximately 2 years following diagnosis. Through careful diagnosis patients with KFD may avoid unnecessary treatment with aggressive chemotherapy and its associated toxicities, which include the risk of secondary malignancies.
