# This corrects the article “Kikuchi-Fujimoto Disease in the Unites States: Three Case Reports and Review of the Literature” in volume 6, e2014001, link: http://www.mjhid.org/article/view/11988

**DOI:** 10.4084/MJHID.2014.023

**Published:** 2014-03-10

Due to a layout error, in the title **Unites** must be canged on **United.** At the first row of the abstract **histiocytic lymphadenitis** must be canged on **histiocytic necrotizing lymphadenitis.**

